# Is the built-environment at origin, on route, and at destination associated with bicycle commuting? A gender-informed approach^☆^

**DOI:** 10.1016/j.jtrangeo.2021.103120

**Published:** 2021-06

**Authors:** Diana Higuera-Mendieta, Pablo Andrés Uriza, Sergio A. Cabrales, Andrés L. Medaglia, Luis A. Guzman, Olga L. Sarmiento

**Affiliations:** a School of Medicine, Universidad de los Andes, Bogotá, Colombia; b Department of Industrial Engineering, Center for Optimization and Applied Probability, Universidad de los Andes, Bogotá, Colombia; c Department of Civil and Environmental Engineering, Grupo de Estudios en Sostenibilidad Urbana y Regional – SUR, Universidad de los Andes, Bogotá, Colombia; d Industrial Engineering Program, School of Exact Sciences and Engineering, Universidad Sergio Arboleda, Bogotá, Colombia

**Keywords:** Bicycle, Commuting, Gender, Generalized additive models, Latin America, Urban health

## Abstract

There is limited evidence on the gender differences and location-specific built-environment factors associated with bicycling in Latin American cities. This study aimed to assess commuting in Bogotá by (1) analyzing the gender-specific trend of the standardized number of bicycle commuters during 2005–2017; and (2) assessing the socio-demographic, community, built-environment and natural factors associated with bicycle commuting stratified by gender. This secondary-data analysis included data from the Household Travel Surveys and Multipurpose Surveys to calculate the number of bicycle commuters per habitant from 2005 to 2017 by gender. We assessed the socio-demographic and built-environment factors fitting generalized additive models stratified by gender using the 2015 Household Travel Survey. Although both women and men increased the standardized number of bicycle commuters, male commuters show a steeper trend than women, evidencing the widening gender gap in bicycle commuting over time. Bicycle commuting was negatively associated with household motor vehicle ownership, steeper terrain slope, longer commute distance, and scarce low-stress roads at trip origin and route. Among women, the availability of bike paths at the trip destination was positively associated with bicycling, while age and being a student were negatively associated with bicycling. Among men, living in areas with the lowest socio-economic status was positively associated with bicycling, while having a driver's license and living close to bus rapid transit stations were negatively associated with bicycling. In conclusion, bicycle and transport infrastructure play different roles in commuting by bicycle by gender and trip stages (origin – route – destination).

## Highlights

• Inclusion of location-based factors unveils potential for transport analysis.
• Methodology could be applied in cities with similar contexts.
• Bicycle commuting in Bogotá has been increasing with marked gender differences.
• Associated factors with bicycle commuting differ by gender.
• The results may support gender-sensitive mobility planning.

## 1 Introduction

Bicycle commuting has multiple health benefits, including the promotion of physical activity (Fishman et al., 2015), the prevention of non-communicable diseases (Taddei et al., 2015), and the reduction of perceived stress (Avila-Palencia et al., 2017). Among women bicycling has been associated with improvements in self-esteem, empowerment, feelings of freedom and autonomy (Lira, 2020). Bicycling is one of the most resource-effective urban transport modes with small space usage, low costs of user-entry and public infrastructure (Pucher and Buehler, 2017; Tranter, 2018). Bicycling also contributes to reducing traffic congestion and car use (Fishman et al., 2014; Wang and Zhou, 2017). Moreover, bicycling is an environmentally and socially sustainable transport mode, contributing to reductions in greenhouse gas emissions (Buehler and Pucher, 2012; Lovelace et al., 2015; Verma et al., 2016). Several governments, public health agencies, and the United Nations have recognized the importance of the bicycle's in achieving the Sustainable Development Goals (The United Nations, 2018).

Despite the multiple benefits of bicycling, the assessment of socio-demographic and built-environment factors encouraging bicycling show different results depending on the study context. Existing evidence is not consistent, mainly because built-environment attributes are related to different domains of cycling behaviors (Yang et al., 2019). However, those findings have provided relevant information for planning and policymaking, such as infrastructure decisions, urban planning, and public health actions (DiGioia et al., 2017; Handy and Xing, 2011; Oliva et al., 2018; Pettit and Dogde, 2014; Rosas-Satizábal and Rodriguez-Valencia, 2019).

Latin American cities (LAC) provide some compelling examples of the rapid growth in bicycle use, including a six-fold increase in Bogotá (Colombia) and a two-fold increase in Santiago (Chile) over a decade (Pucher and Buehler, 2017). This has motivated research throughout the region on the determinants of bicycle commuting, with recent studies in Colombia, Chile, and Brazil (Becerra et al., 2013; Rosas-Satizábal and Rodriguez-Valencia, 2019). Despite the increase in research and bicycle use, the study regarding built-environment factors, such as the existence and location of bicycle infrastructure and accessibility to public transportation, accounted for gender differences in cycling. Given that women account for a minor fraction of bicycle trips (Rios et al., 2015), understanding this gap is crucial to plan, design, and build better cities to promote and encourage more equitable bicycle commuting.

Since travel behavior is heavily influenced by gender (Garrard et al., 2007), research that includes such perspective contributes to a more inclusive transport planning that supports the reduction of inequalities for women (Prati, 2018). These gender-informed approaches are particularly relevant in LAC contexts, where gender gaps are more pronounced than in high-income countries (World Economic Forum, 2020). In LAC, women's participation in labor markets is lower (Allen et al., 2018), and the proportion of women who suffered intimate partner physical and sexual violence is higher than in the Global North (Allen et al., 2018).

Gender differences in factors associated with bicycle commuting and the built-environment influence have limited studies in LAC, and gender is usually limited within existing studies to being a control variable in regression models (Guerra et al., 2018; Preston and McLafferty, 2016; Rodriguez-Valencia et al., 2019). The bulk of literature on this topic comes from high-income countries, indicating that increasing bicycle share does not necessarily increase women's representation (Aldred et al., 2016; Prati, 2018). Furthermore, most of the existing analyses addressing bicycle commuting have been focused on the built-environment factors at the origin of the trip. Only a few have considered infrastructure variables at both the origin, route and destination (Adlakha and Parra, 2020; Cole-Hunter et al., 2015; Heinen et al., 2010; Oliva et al., 2018).

It is crucial to support policies promoting a more inclusive cycling environment in LAC. The social-ecological framework, in which several factors influence bicycle commuting at different levels (i.e., individual socio-demographic, community, built-environment, natural and policy factors) (Acheampong and Siiba, 2018; Sallis et al., 2002) could serve as a tool by filling the knowledge gap about gender-specific factors associated with bicycle commuting and considering origin, route, and destination characteristics. Thus, the main objective of this study is to examine the existing gender differences in bicycle commuting in Bogotá by (1) describing the trend of the standardized number of bicycle commuters during the period 2005–2017 stratified by gender; and (2) assessing the socio-demographic, community, built-environment and natural factors associated with bicycle commuting stratified by gender.

We hypothesized a higher increase in biking trend in males compared to females. In addition, we hypothesized a differential association of socio-demographic, community, and built-environment factors with bicycle commuting by gender. Specifically, among men, we hypothesized negative associations between bicycle commuting and socio-demographic factors (i.e., age, income, employment, having a driver's license, vehicle ownership, and trip distance) while positive associations with some built-environment factors (i.e., bicycle infrastructure at the destination) and negative association with public transportation accessibility. Among women, we hypothesized negative association with age, and positive associations with safety perception, and built-environment factors such as bicycle infrastructure and low-stress conditions in the road.

This paper is structured as follows. Section 2 provides a literature review on bicycle commuting and gender-informed factors associated with bicycle commuting. Section 3 presents the methodology including the study setting, data sources, and statistical analysis. Section 4 includes the results of the trend analysis and the factors associated with bicycle commuting. Section 5 features the discussion of the results considering existing literature, address the limitations of the study, suggests future work, and provides public policy recommendations. Finally, Section 6 presents conclusions.

## 2 Literature review

**Fig. 1:**
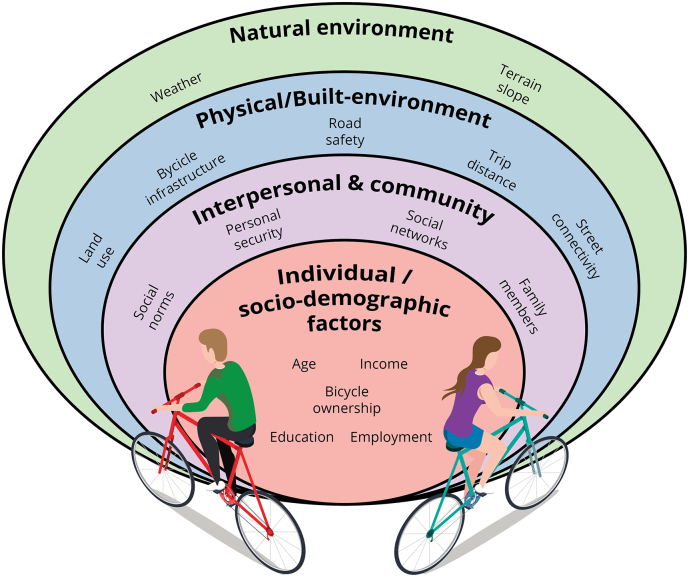
Socio-ecological model for bicycle commuting. The interrelationship between several factors influence bicycle commuting at different levels. Own elaboration, based on (Acheampong and Siiba, 2018; Badland et al., 2013; Sallis et al., 2002).

The social-ecological model (Fig. 1), has been widely used for the study of health behaviors and health promotion and suggests that behaviors are determined by multiple levels of factors, beginning by individual factors, expanding outward to community, physical and natural environments (Handy and Xing, 2011; Sallis et al., 2002). The individual level includes socio-demographic characteristics of the person, such as age, occupation, and income. The community-level often refers to social norms, the tacit behaviors that are considered appropriate for a group. The physical and natural level refers to the built-environment factors or characteristics of the natural environment that could facilitate or hinder the behavior. Empirical results reiterate that examining different levels, such as the socio-demographic and built-environment, would contribute to understanding bicycle commuting (Acheampong and Siiba, 2018; Badland et al., 2013; Sallis et al., 2013). Evidence of the underlying socio-demographic, community, built-environment and natural factors associated with bicycling in cities has been generated across multiple global regions, with limited information in the Latin American region (Pucher et al., 2010; Yang et al., 2019).

At the individual level, the relationship between age and income levels with bicycling have been studied showing mixed results. While some authors identified a negative relationship with age, finding that older people are less likely to use bicycles for transport (Heinen et al., 2010; Pucher et al., 1999; Zacharias, 2005), others have not found evidence of such a relationship (Heinen et al., 2010; Kitamura and Mokhtarian, 1997; Wardman et al., 2007). Similarly, the relationship between income and bicycle commuting remains unclear, with studies showing mixed results in a variety of settings worldwide (Heinen et al., 2010). Employment and occupation also affect the decision to commute by bicycle, with evidence indicating that having a part-time job is positively associated with bicycle commuting (Bonham and Wilson, 2012; Boumans and Harms, 2004). Additionally, the available evidence shows that ownership of a motorized vehicle or having a driver's license (Emond and Handy, 2012; Handy et al., 2014; Heinen et al., 2010; Orsini and O'brien, 2006; Pucher et al., 1999), and longer trip distances (Cole-Hunter et al., 2015; Dickinson et al., 2003; Parkin et al., 2008; Stinson and Bhat, 2004; Timperio et al., 2006) diminish the likelihood of commuting by bicycle. Furthermore, research conducted in multiple Latin American cities has found that bicycle ownership increases the likelihood of commuting by bicycle (Cervero et al., 2009; Florindo et al., 2018; Kienteka et al., 2014).

At the community level, the evidence suggests that having relatives who use the bicycle offer support through bicycling and are more likely to commute by bicycle (Handy and Xing, 2011). Contrary, a social pressure for owning a car (Sigurdardottir et al., 2013), a lower status associated to use bicycle (Acheampong and Siiba, 2018), and poor personal security conditions hinder the probability of bicycle commuting (Arellana et al., 2020; Bernardo and Bhat, 2014; Singleton and Wang, 2014).

At the physical level, research examining the connection between built-environment factors such as mixed-use settings (Cervero, 1996; Cervero and Kockelman, 1997; Litman and Steele, 2018), bicycle facilities (Heinen et al., 2010; Rodriguez-Valencia et al., 2019), the presence of bike paths (Cervero et al., 2009; Florindo et al., 2018; Oliva et al., 2018; Rodriguez-Valencia et al., 2019), block size and street density (Cervero et al., 2009; Oliva et al., 2018) have found a positive association with bicycling. In contrast, increased public transport accessibility diminishes the likelihood of bicycling (Cole-Hunter et al., 2015; Dickinson et al., 2003; Parkin et al., 2008; Stinson and Bhat, 2004; Timperio et al., 2006). As well as road characteristics that create stressful conditions for the bicyclists like those summarized by the Level of Traffic Stress (LTS) (Furth et al., 2016; Huertas et al., 2020; Mekuria et al., 2012).

The available evidence has shown a negative association between bicycle commuting, public transport use (Cervero et al., 2009; Florindo et al., 2018; Oliva et al., 2018) and motor vehicle ownership (Cervero et al., 2009; Florindo et al., 2018; Oliva et al., 2018; Rodriguez-Valencia et al., 2019). Studies examining how land use mix and the age of the commuter may influence the likelihood of bicycling (Cervero et al., 2009; Florindo et al., 2018) are inconclusive. Finally, poor road safety can further diminish the probability of bicycle commuting (Arellana et al., 2020; DiGioia et al., 2017; Dill and McNeil, 2017; Pettit and Dogde, 2014).

At the natural level, research has found that steep terrain significantly reduce the likelihood of bicycling (Hunt and Abraham, 2007; Moudon et al., 2005; Parkin et al., 2008; Rietveld, 2000; Rodríguez and Joo, 2004; Stinson and Bhat, 2004).

The available evidence suggests that men are more likely to use bicycles for transport than women (Aguilar-Farias et al., 2019; Cervero et al., 2009; Heinen et al., 2010; Kienteka et al., 2014; Reis et al., 2013; Sá et al., 2016). Some studies have addressed such gender gap in bicycle commuting in their analyses (Carroll et al., 2020; Heesch et al., 2012; Preston and McLafferty, 2016; Singleton and Goddard, 2016). Assessments in the Global North have identified that short distances, carrying children, and more risk aversion in women might explain why they commute by bicycle less than men (Aldred et al., 2016). However, these studies are primarily ecological or descriptive. Additionally, several qualitative approaches addressing modal choice, including bicycle, in women of LAC have identified sexual harassment, safety concerns, and hostility perception towards women in the streets as critical factors (Allen et al., 2018; de la Paz Díaz Vázquez, 2017; Lira, 2020; Montoya-Robledo et al., 2020; Orozco-Fontalvo et al., 2019). A number of authors have identified a stronger preference for segregated bicycle infrastructure among women (Aldred et al., 2016; Garrard et al., 2007; Lusk et al., 2014). Nonetheless, there is limited evidence on whether the location of this infrastructure (origin, route, or destination) plays a particular role in bicycle commuting or whether such relationship is differential by gender.

Differential effects of socio-demographic factors by gender have been recently addressed in Dublin, Ireland, at a district level. They have identified a higher negative effect of income on bicycle commuting in women than in men (Carroll et al., 2020). In Oregon, US, more educated women were more likely to cycle than their less-educated counterparts, while no relationship was found in men. Additionally, men with lower income were more likely to engage in bicycle commuting than men with higher-income, and no such correlation was found in women (Singleton and Goddard, 2016). Studies assessing accessibility to employment and education among bicycle users in Bogotá found marked differences in bicyclists based on class and gender (Rosas-Satizábal et al., 2020). Thus, indicating the importance of addressing this topic with a gender informed approach in the region. Those works demonstrate the importance of socio-demographic and built-environment variables in bicycle use, showing the complexity of these relationships. To our knowledge, no empirical work has tackled this complexity and examined the role of socio-demographic, community, built-environment and natural characteristics at the origin, route and destination of the trip in a developing city in the Global South.

Our study adds to current knowledge by filling the gap regarding the simultaneous assessment of socio-ecological factors associated with bicycle commuting and its differential role by gender in Latin America by using individual cross-sectional data and location-specific information of the trips (origin, route, and destination).

## 3 Methods

### 3.1 Study setting

Bogotá, the capital city of Colombia, is characterized by social, spatial and transport-related inequalities (Guzman and Bocarejo, 2017). The city is a dense metropolis with 7.2 million inhabitants (DANE, 2018a) in an urban area of 380 km^2^. The city has the most extensive bicycle infrastructure in Latin America, with 551 km of bike paths (SDM, 2020a) and more than 600,000 bicycle trips per day (SDM, 2015). Policies promoting bicycle use in Bogotá has been the result of advocacy efforts from citizens (Rosas-Satizábal and Rodriguez-Valencia, 2019) and committed local administrations with the provision of infrastructure for non-motorized transport modes (Ardila and Menckhoff, 2002).

The city has experienced a transformation in which multimodality, including bicycle use, has been promoted (Montezuma, 2005). After an initial large investment in bicycle infrastructure in the late 1990s, the city has constructed a network of dedicated bicycle lanes and pathways spanning 551 km, encouraging bicycle commuting and achieving an increase in bicycling's modal share from 0.58% in 1996 to 9.10% in 2017 (Rosas-Satizábal and Rodriguez-Valencia, 2019), and positioning the city among the top 10 most bike-friendly cities in the world (Yorck von Wartenburg, 2019). However, trips by women account for only 25.8% of such trips (Rosas-Satizábal et al., 2020).

After a significant investment in bicycle infrastructure between 2000 and 2005, city administrations have focused on different aspects of existing cycling infrastructure (Rosas-Satizábal and Rodriguez-Valencia, 2019). The efforts have included improving the experience of current cyclists (i.e., paving bike-paths) and increasing ridership (i.e., increasing access to cycling infrastructure in low SES areas) (Rosas-Satizábal and Rodriguez-Valencia, 2019). Recently, the city administration has implemented a comprehensive policy framework called *Plan Bici* to promote bicycling as the preferred transportation mode (SDM, 2016a; 2016b). The Plan Bici compiles various policies and programs targeting multiple population groups and integrating several sectors, including education, mobility, and security. More specifically, regarding gender inequalities, alliances between Bogota's Secretary of Women and Secretary of Mobility have allowed to act collectively on security perception in women who cycle and promote raising awarenes on sexual harrasment in public transportation (Moscoso et al., 2020).

During the COVID-19 pandemic, Bogotá promoted bicycle commuting by temporarily closing 85 km of roads to motorized vehicles, converting them into bicycle paths, and increasing the current network's connectivity (SDM, 2020b). The city government promoted bicycle commuting not only as a sustainable transport mode but also to reduce exposure to SARS Cov-2 by decreasing the number of close contacts that a commuter might experience in public transport. More than 340,000 commuters were recorded during the first stages of the city's lock-down in March and April of 2020. Among essential workers surveyed during these two months, 68% reported switching to the bicycle as their main mode of transport. However, gender differences remained as only 34% were female (SDM, 2020c).

### 3.2 Analytical approach

Our approach included two steps. First, we analyzed trends in the makeup of the bicycle commuter population by gender in Bogotá for 2005–2017 using a Mann-Kendall test to evaluate whether there was a monotonic trend. Second, we assessed the socio-demographic, community, built-environment and natural factors at origin, route, and destination associated with bicycle commuting by fitting multivariate generalized additive models (GAM).

We performed all the data processing and analyses using R (R Core Team, 2018) along with the following R packages: *tidyverse* (Wickham et al., 2019) for data wrangling and visualizations; *sf* (Pebesma, 2018), *stplanr* (Lovelace and Ellison, 2019), and *raster* (Hijmans, 2020) for spatial data processing; and *mgcv* (Wood, 2017) for statistical modeling.

### 3.3 Data sources

#### 3.3.1 Trend analysis

We used five official surveys: Household Travel Surveys from 2005, 2011, and 2015 (SDM, 2015; 2011; 2005); and Encuestas Multipropósito (in English: Multipurpose Surveys) from 2014 and 2017 (SDP, 2017; 2014). We extracted data from each survey on the number of individuals who reported using the bicycle as their usual transport mode for trips to work or study. We calculated the number of bicycle commuters per 100,000 inhabitants in Bogotá using official population projections for each available data year.

#### 3.3.2 Factors associated with bicycle commuting

To assess the associations between socio-demographic, community, built-environment and natural factors with bicycle commuting by gender, we used the 2015 Household Travel Survey (SDM, 2015). The sample included 28,213 households, comprised of 91,765 individual respondents who reported 147,251 trips. The respondents were asked about their socio-demographic characteristics (e.g., gender, main occupation, household motor vehicle ownership) and all trips conducted the day before the survey with their geocoded origin and destination. According to the survey definition, a trip is the combination of modes used to get from one place to another. Every change of transport mode (e.g., bicycling to public transport) represents a stage within a trip. For our analysis, we only considered the following trips: 1) trips where the origin and destination fall within the official boundaries of Bogotá, 2) trips for commuting purposes (i.e., travel to work or study) on business days, 3) trips reported by individuals older than 14 years of age, and 4) the first trip completed for each individual as only 3.9% of the individuals reported more than one trip. The final sample consisted of 16,495 trips (with an equal number of individuals), including the geocoded origin and destination of a trip, the trip's purpose, and the transport mode at each stage.

Five secondary data sources were used for the generation of community, built-environment and natural variables, including 1) the local geospatial office IDECA (Unidad Administrativa Especial de Catastro Distrital, 2018), 2) the official record of thefts and robberies of Bogotá (Policía Nacional de Colombia, 2018), 3) the compilation of collisions used in Carvajal et al. (Carvajal et al., 2020), 4) the Japanese Aerospace Exploration Agency (JAXA) (Tadono et al., 2016), and 5) the level of traffic stress-based classification of the roads of Bogotá (Huertas et al., 2020)**.**

### 3.4 Geographic Information System (GIS) analysis zones

**Fig. 2:**
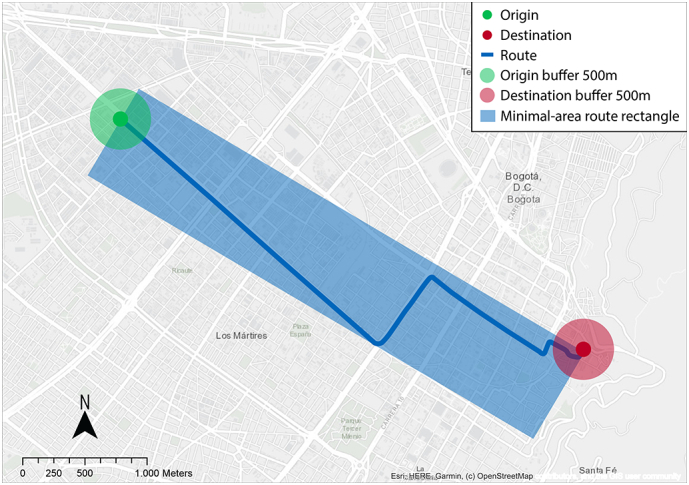
Illustration of GIS analysis zones. The example depicts trip zones of geocoded bicycle commuters trip including origin, route, and destination within the minimal-area rectangle that encloses the shortest path. Own elaboration.

To assess the built-environment factors associated with bicycle commuting, we used GIS in three zones for each respondent's reported trip: origin, route, and destination. First, for each geocoded origin and destination, we generated a 500-meter radial buffer. The buffer's size was selected based on what other studies in Bogotá have used to ensure comparability (Rodriguez-Valencia et al., 2019). Second, we estimated the respondent's route by calculating the shortest path between the origin and destination (Luxen and Vetter, 2011) over the road network. Third, as bicyclists may not strictly follow the shortest path, to characterize the built-environment along the route, we defined the minimal-area rectangle that encloses the shortest path (Cole-Hunter et al., 2015). Fig. 2 shows an example of the three GIS analysis zones for one specific trip.

### 3.5 Statistical analysis

#### 3.5.1 Trend analysis

We assessed the changes in the standardized number of bicycle commuters per 100,000 inhabitants for 2005–2017 stratified by gender. We conducted a Mann-Kendall test to assess whether there was a monotonic trend in the standardized number of bicycle commuters per 100,000 inhabitants between 2005 and 2017.

#### 3.5.2 Factors associated with bicycle commuting

First, we conducted a bivariate analysis by comparing bicycle commuters vs. non-commuters (based on the most recent trip reported in the travel survey) according to their socio-demographic, community, built-environment and natural factors at origin, route, and destination. We described the proportion of bicycle commuting by socio-demographic factors adjusting for the sample-design weights. For the categorical variables, we compared both groups using a chi-squared test with Rao & Scott's design-adjustment (Rao and Scott, 1984). For the continuous variables, we compared medians using the design-based Kruskal-Wallis test across bicycle commuting groups.

Second, we fitted multivariate Generalized Additive Models (GAM) to assess the socio-demographic, community, built-environment and natural factors associated with bicycle commuting. GAM can be seen as a flexible extension of the more common Generalized Linear Models that model nonlinear relationships by not imposing monotonic relationships between the predictors and the dependent variable (Hastie and Tibshirani, 1986; Wood, 2017). To quantify the association of socio-demographic and built-environment variables with the odds of bicycle commuting, we modeled log odds of bicycle commuting as a function of parametric factors and smooth nonparametric functions as follows:

(1) $$\mathrm{logit}\left(p\right)=\log\left(\frac{p}{1-p}\right)=\beta_{0}+\sum_{j=1}^{J}\beta_{j}x_{j}+\sum_{k=1}^{K}s\left(x_{k}\right)$$

where the variables *x*_*j*_ modeled as parametric factors appear with their respective *β*_*j*_ coefficient; and variables *x*_*k*_ modeled as smooth nonparametric functions (i.e., age, commute distance, land use mix, the proportion of low and extremely-high LTS roads) appear wrapped in expressions of the format *s*(*x*_*k*_) (Eqn 1).

We fitted six models, one for each combination of gender (male and female) and analysis zones (origin, destination, and route). We checked for multicollinearity using the Variance Inflation Factor (VIF) and excluded variables with VIF values greater than 3.0.

The dependent variable was bicycle commuting, a dichotomous variable that takes 1 if the bicycle was used to complete at least one stage of the trip and 0 otherwise. The independent variables included socio-demographic factors and built-environment factors.

##### 3.5.2.1 Socio-demographic factors

Socio-demographic factors included gender, age, type of driver's license (i.e., motorcycle, other vehicles), and main occupation (i.e., student, employed, other). Household characteristics included socio-economic status (SES) of the neighborhood, using the official socio-economic stratification system of the country based on the physical characteristics of the dwelling and its surroundings, which correlates well with household income (Cantillo-García et al., 2019) (i.e., very-low to low, lower-middle, middle to high); and motor vehicles ownership.

##### 3.5.2.2 Community factors

Community factors included safety and security conditions. Safety was measured using the number of reported collisions involving bicycle users between 2016 and 2017 (Carvajal et al., 2020). We used the number of criminal reports for measuring security, including thefts and felonies, within each urban planning zone (UPZ) in 2015 (Policía Nacional de Colombia, 2018). The UPZs are the official planning and data reporting zones of Bogotá's public administration (Alcaldía Mayor de Bogotá, 2004). These are used for planning urban development at the zonal level and are bounded by roads and natural borders (Guzman and Bocarejo, 2017). We used areal-weighted interpolation to estimate the number of felonies per inhabitant in each GIS analysis zone. Bogotá has a total of 113 UPZ with an average area of 3.6 km^2^.

##### 3.5.2.3 Built-environment factors

Built-environment factors included land use, bicycle infrastructure, public transport accessibility, road network characteristics, commuting distance. We measured the land use mix within a zone using an entropy index (Sallis et al., 2013), which ranges from 0 (homogeneous) to 1 (heterogeneous). Bicycle infrastructure comprises the total length of bike paths (km) and the number of bicycle parking facilities. Public transport accessibility included the number of Bus Rapid Transit (BRT) stations and bus stops. Road network characteristics included the proportion of roads classified at different Levels of Traffic Stress (LTS). The proportion of roads classified as low LTS and extremely high LTS were calculated using available road network data for Bogotá (Huertas et al., 2020). Road segments labeled with low LTS are characterized by narrow streets, few lanes, low speed, low traffic density, low traffic flow, and low congestion. Also, these road segments show no presence of public transport lines and a lack of bicycling infrastructure. On the other hand, road segments labeled with extremely high LTS are characterized by wide roads, an average number of lanes, high vehicle speed, high traffic density, high traffic flow, high congestion, lack of bicycling infrastructure, and presence of public transport lines. Bicycle infrastructure, public transport and accessibility variables along the route were normalized using the trip distance (km).

##### 3.5.2.4 Natural factors

Natural factors included the average terrain slope that was calculated using the Digital Surface Model developed by the Japanese Aerospace Exploration Agency (JAXA) with a 30-m mesh resolution (Tadono et al., 2016).

## 4 Results

### 4.1 Trend analysis

**Fig. 3:**
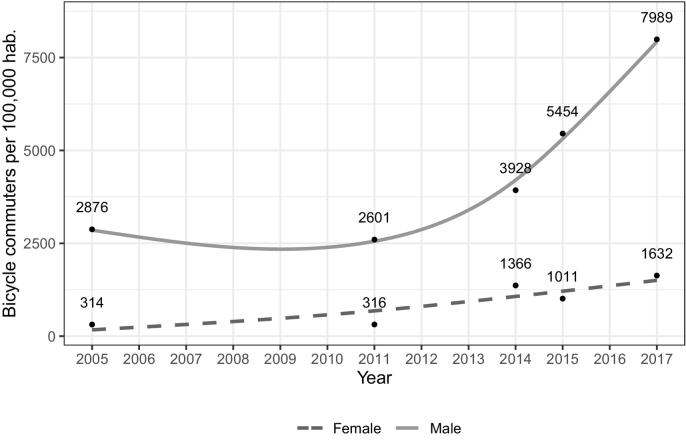
Estimated number of bicycle users per 100,000 inhabitants in Bogotá for the total population and stratified by gender 2005–2017. Smoothed trendlines with data from 2005, 2011, 2014, 2015, and 2017.

We found an increase in the number of bicyclists compared to the gross population growth that differed by gender (Fig. 3). For males, the increase from 2005 to 2017 was of 5113 bicycle users per 100,000 inhabitants (slope: 426.08 and 177.8% increase) [*p* < 0.01]; while for females the increase from 2005 to 2017 was of 1318 bicycle users per 100,000 inhabitants (slope: 109.83 and 419.7% increase) [p < 0.01]. There was evidence of a significant monotonic trend during the whole period for both series, despite the modest reduction observed during the years 2005–17. The gap between females and males has widened with time.

### 4.2 Socio-demographic, community, built-environment and natural factors associated with bicycle commuting

Bicycle commuters were mainly male (82.8%) aged 18–29 years old (40.8%), employed (80.7%), with no driver's license (70.0%), living in low or very-low socio-economic status households (58.3%), and with no ownership of motorized vehicles in the household (80.7%) (Table 1). The average distance traveled by a bicycle commuter was 6.88 km.

**Table 1:** Socio-demographic characteristics of bicycle and non-bicycle commuters in Bogotá (2015).

Variable	Bicycle commuter		Non-bicycle commuter		p-value^b^
	n = 768		n = 15727		
	Frequency	(%)^a^	Frequency	(%)^a^	
Gender					
Male	647	(82.82%)	8452	(53.63%)	<0.001
Female	121	(17.18%)	7275	(46.37%)	
Age					
14–17	50	(4.82%)	1734	(10.64%)	0.003
18–29	254	(40.77%)	4740	(31.35%)	
30–49	314	(39.08%)	6097	(39.46%)	
50–64	129	(13.52%)	2731	(16.29%)	
≥ 65	21	(1.80%)	425	(2.27%)	
Main occupation					
Student	125	(14.03%)	3816	(24.34%)	
Employed	608	(80.68%)	11070	(70.18%)	
Other^d^	35	(5.29%)	841	(5.49%)	
Driver's license					
For motorcycle	37	(4.98%)	884	(6.71%)	
For other vehicles^c^	178	(25.07%)	4586	(29.50%)	
No license	553	(69.95%)	10257	(63.80%)	
Household socio-economic status^e^					
Very-low to low	428	(58.34%)	8038	(46.91%)	0.025
Lower-middle	267	(31.27%)	5607	(36.23%)	
Middle to high	73	(10.39%)	2082	(16.86%)	
Motor vehicles ownership in the household	187	(19.26%)	6559	(43.01%)	<0.001
Average commute distance [km], Mean (SD)	6.88	(0.53)	8.78	(0.09)	0.003

a All the proportions were weighted by the expansion factors of the sample.

b Chi-square test with Rao & Scott design- adjustment for categorical variables and design-based Kruskall-Wallis for continuous.

c Includes: driver's license for car, and driver's license for other unspecified vehicles.

d Includes: Job-seeking, Housekeeping, Retired, and other unspecified activity.

e Very-low to low = 1, 2; Lower-middle = 3; Middle to high = 4, 5, 6.

**Fig. 4:**
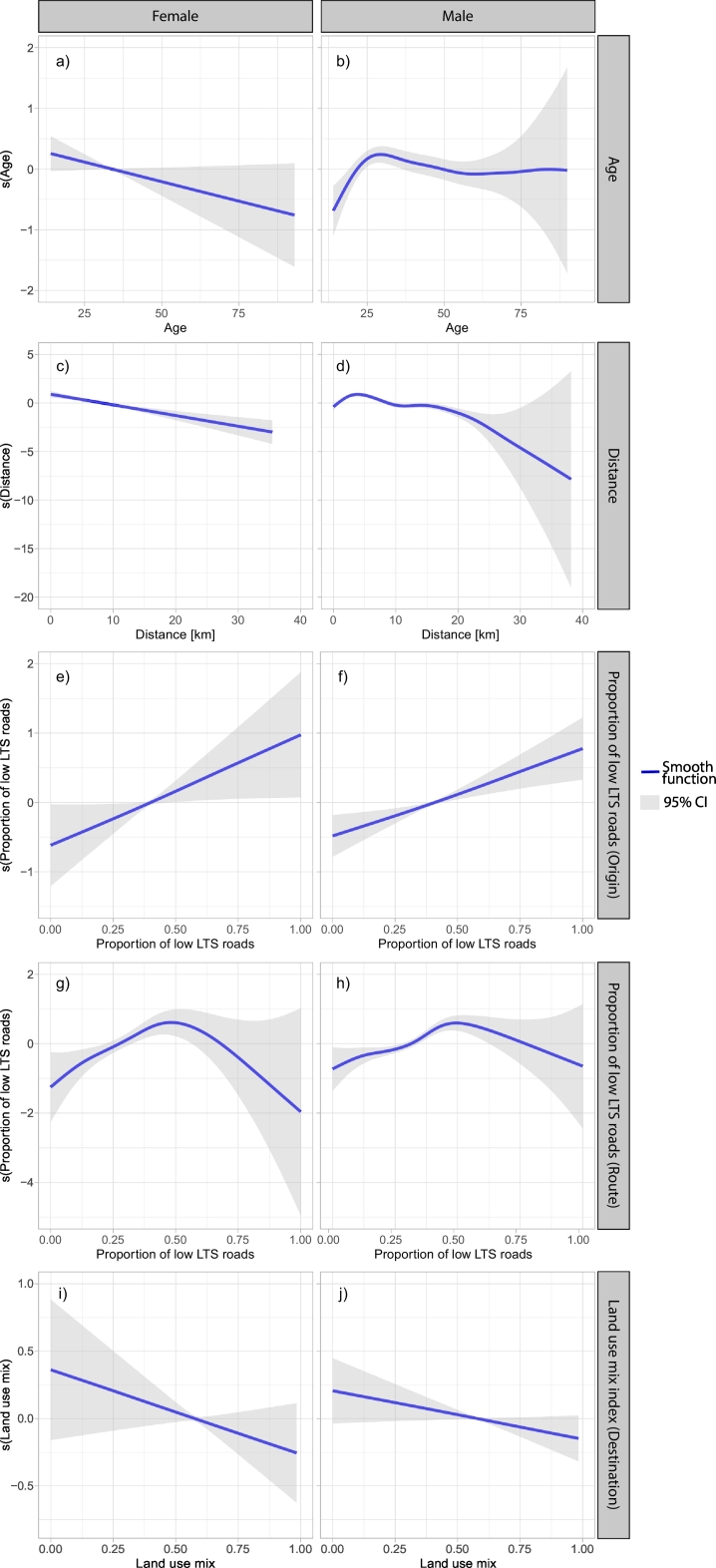
Association of socio-demographic and built environment factors with bicycle commuting among women and men. Nonparametric splines (s) and 95% confidence intervals (gray bands). There is a linear relationship between bicycle commuting and age, distance, proportion of low LTS roads and land use mix in females while for males it is observed that there is change in the direction of the association between bicycle commuting and age after 25 years old and distances longer than 4 km.

Table 2 summarizes the results of the stratified GAM models for females and males, and Fig. 4 shows smooth functions for the variables included as nonparametric splines.

**Table 2:** Socio-demographic and built-environment factors associated with bicycle commuting in Bogotá 2015 from the six fitted models, one for each combination of gender (male and female) and analysis zones (origin, route, and destination).

	Origin				Route				Destination			
	Females		Males		Females		Males		Females		Males	
**Parametric terms:**	OR	(95%CI)	OR	(95%CI)	OR	(95%CI)	OR	(95%CI)	OR	(95%CI)	OR	(95%CI)
***Socio-demographic factors***												
Occupation												
Other (ref)	1.00		1.00									
Student	0.31	(0.14–0.71)	0.78	(0.46–1.34)								
Employed	0.93	(0.5–1.74)	1.11	(0.71–1.76)								
Driver's license												
No license (ref)	1.00		1.00									
Motorcycle	1.16	(0.27–4.92)	0.53	(0.36–0.78)								
Other vehicles	1.02	(0.56–1.88)	0.51	(0.41–0.64)								
Household socio-economic status												
Very-low to low (ref)	1.00		1.00									
Lower-middle	0.83	(0.52–1.3)	0.76	(0.55–0.97)								
Middle to high	1.53	(0.76–3.11)	0.9	(0.54–1.26)								
Motor vehicles ownership in the household	0.58	(0.36–0.91)	0.53	(0.3–0.76)								
***Community factors***												
*Safety*												
Collisions	1.00	(0.99–1.01)	1.01	(1.01–1.01)	1.00	(0.98–1.02)	1.00	(0.99–1.00)	1.00	(0.99–1.01)	1.01	(1.00–1.01)
*Security*												
Felonies per inhabitant	1.00	(1.00–1.00)	1.00	(1.00–1.00)	Ɨ		Ɨ		1.00	(1.00–1.00)	1.00	(1.00–1.00)
***Built-environment factors***												
*Bicycle infrastructure*												
Bicycle parking facilities	0.86	(0.6–1.24)	0.96	(0.8–1.12)	Ɨ		Ɨ		0.96	(0.83–1.11)	1.02	(0.96–1.09)
Bike paths length	0.93	(0.76–1.14)	0.96	(0.86–1.06)	1.16	(0.82–1.64)	1.06	(0.91–1.23)	1.26	(1.05–1.51)	1.01	(0.92–1.1)
*Public transport accessibility*												
Count of BRT^a^ stations	0.85	(0.6–1.21)	0.82	(0.66–0.98)	Ɨ		Ɨ		0.79	(0.60–1.04)	0.85	(0.75–0.96)
Count of bus stops	1.00	(0.97–1.03)	0.99	(0.98–1)	Ɨ		Ɨ		0.99	(0.97–1.02)	0.99	(0.98–1.01)
***Natural factors***												
Average terrain slope	0.84	(0.78–0.89)	0.93	(0.91–0.95)	0.82	(0.75–0.9)	0.88	(0.85–0.91)	0.98	(0.93–1.02)	0.97	(0.95–0.99)
**Smooth terms:**	E.d.f	p-value	E.d.f	p-value	E.d.f	p-value	E.d.f	p-value	E.d.f	p-value	E.d.f	p-value
***Socio-demographic factors***												
s(Age)	0.77	0.04	4.30	<0.01								
s(Commute distance)	1.01	<0.01	6.61	<0.01								
***Built-environment factors***												
*Land use*												
s(land use mix index)	0.00	0.68	0.00	0.53	Ɨ		Ɨ		0.67	0.08	0.77	0.04
*Road network*												
s(Proportion of Low LTS^b^ roads)	0.86	0.02	1.02	<0.01	3.05	<0.01	4.21	<0.01	0.00	0.91	0.00	0.50
s(Proportion of Extremely-high LTS^b^ roads)	0.00	0.38	0.00	0.69	0.00	0.69	0.64	0.10	0.36	0.20	0.00	0.58
**Deviance explained**	9.78%		9.86%		9.31%		10.30%		6.13%		9.23%	

Link function: Logit.

s() refers to a spline function.

All the models were adjusted by socio-demographic factors.

a Bus Rapid Transit system TransMilenio.

b Level of Traffic Stress.

Ɨ Not included in the model.

##### 4.2.1.1 Factors associated with bicycle commuting regardless of gender

Among the examined socio-demographic factors, we found that ownership of motorized vehicles was negatively associated with the propensity to commute by bicycle. Regarding built-environment characteristics, a steeper average terrain slope was negatively associated with bicycle commuting at origin and route. When examining characteristics of roadways near the origin point of trips, as the proportion of low LTS road segments near trip origin locations increases, the propensity for bicycle commuting increases linearly (Fig. 4e, and f). Along the route of the trip, the relationship between bicycling and proportion of low LTS roads had a nonlinear behavior. Specifically, there was a positive linear relationship with bicycle commuting until the proportion of low LTS roads reaches 50%; when the proportion of low LTS roads exceeds 50%, the propensity for bicycle commuting decreases linearly, although the confidence intervals are too wide to draw reliable conclusions from this observed decline (Fig. 4g, and h). When examining characteristics of roadways near the destination point of trips, Higher land use mix was negatively associated with the propensity of bicycle commuting.

##### 4.2.1.2 Factors associated with bicycle commuting in women

Among the considered socio-demographic factors, we found that being a student was negatively associated with bicycle commuting among women, whereas as age increases, the propensity to commute by bicycle decreases linearly (Fig. 4a). As the commute distance of female respondents increases, levels of bicycle commuting decline significantly (Fig. 4a). Among built-environment factors, the length of bike paths near the trip destination was positively associated with bicycle commuting for women, but the same was not found for length of bike paths on route and at trip origin. We did not find a statistically significant association between bicycle commuting, household SES, and public transport accessibility among female respondents.

##### 4.2.1.3 Factors associated with bicycle commuting in men

We found an increase in the probability of bicycle commuting among men under the age of 25 and a slight steady decrease after this point as the age of male respondents increased (Fig. 4b). Additionally, men living in a household categorized as lower-middle SES had a lower tendency for bicycle commuting than men living in households with very low SES. Possessing a driver's license for motorcycles or other vehicles was negatively associated with bicycle commuting among men. Bicycle commuting among men was positively associated with commute distances less than 5 km and negatively associated with longer distances (Fig. 4b). When considering built-environment characteristics at the trip origin and destination locations, the number of nearby BRT stations was negatively associated with bicycle commuting among men. Within all analysis zones, the propensity of bicycle commuting was negatively associated with the proportion of extremely high LTS roads. Steeper average terrain slope of roadways at origin, on route, and at destination was negatively associated with bicycle commuting. Other bicycle infrastructure variables, such as the number of bus stops, safety measures, and security conditions were not found to be significantly associated with bicycle commuting among respondents of either gender or within any of the GIS analysis zones.

## 5 Discussion

This study showed that over twelve years (2005–2017), female and male commuters in Bogotá significantly increased their bicycle commuting rate. However, male commuters show a steeper trend compared to women evidencing the widening gender gap in bicycle commuting. Household motor vehicle ownership, steeper terrain slope, longer commute distance, and a limited number of low-stress roads at trip origin and on the route of the trip were negatively associated with bicycle commuting. Among women, the availability of bicycle paths around their trip destination was positively associated with bicycle commuting, while higher age and being a student were negatively associated. Among men, living in the lowest SES neighborhoods was positively associated with bicycle commuting, while having a driver's license and living close to a BRT station were negatively associated with bicycle commuting.

The study results of the trend analysis are consistent with other studies. Specifically, the observed increasing trend in the proportion of workers commuting by bicycle is consistent with results of studies conducted in the United States (Pucher et al., 1999), Canada (Pucher, 2005), the Netherlands (Harms et al., 2014), and Brazil (Sá et al., 2016). This trend towards increasing bicycle commuting in Bogotá can been explained as being the result of a long process of infrastructure investments, political leadership, clear city-planning documents where bicycle commuting is explicitly named as a goal, a bicycle culture, and active advocacy groups (Rosas-Satizábal and Rodriguez-Valencia, 2019). Bogotá's continuous efforts to promote bicycle use rely not only on political will but also on including specific goals regarding bicycle use in the city's Plan Maestro de Movilidad (Bogotá's Mobility Master Plan). This plan was first developed in 2006, then updated in 2018, adding goals for the year 2030 (Secretaría Distrital de Movilidad, 2019).

Our study expands on the available literature by adding a stratified analysis of this trend towards increased bicycle commuting by gender. Bicycle commuting has indeed increased in both female and male populations within the twelve-year period of analysis. However, higher rates of growth in men commuting by bicycle reveal an important gender disparity and opportunity to identify strategies for increasing female bicycle commuting. The significant increase in female bicycle commuters might show evidence of the women's social movement promoting bicycle use among women. In 2019, the Women's Secretariat of Bogotá convened 15 women's bicycle collectives with the aim of increasing bicycle use among women in Bogotá. Some of these collectives have been active since 2000 (Alcaldía Mayor de Bogotá, 2019). However, the increasing gender gap might be rooted in the underlying unequal distribution of labor (Montoya-Robledo et al., 2020) and previous administrations' focus on providing infrastructure for long, linear trips that men usually do (Allen et al., 2018; Prati, 2018; Singh, 2020). Since women tend to have multiple trips in a day, mostly related to household maintenance and family-care, local governments should take intersectoral steps to understand the mobility of care (Montoya-Robledo et al., 2020) and also promote a more evenly distribution of household labor in LAC (Allen et al., 2018).

We identified two factors that were negatively associated with bicycle commuting, regardless of gender and location, namely that ownership of motorized vehicles and an increasing average terrain slope were negatively associated with bicycle commuting. Our findings related to vehicle ownership are aligned with and supported by pre-existing evidence (Paleti et al., 2012; Sá et al., 2016). Additionally, this finding shows that bicycles may be a temporary commuting option as commuters work to acquire a motorized mode of transport. In the context of the rapid growth in the ownership of motorized vehicles, school-based programs such as al Colegio en Bici (“To school by bike”) and biking programs on the Sunday Ciclovia in Bogotá might increase interest in alternative means of transport during adulthood (Rosas-Satizábal et al., 2020). Regarding our finding that increasing average terrain slope is negatively associated with bicycling, previous research has also found that the probability of bicycling decreases as the terrain slope becomes steeper (Hunt and Abraham, 2007; Parkin et al., 2008; Rietveld and Daniel, 2004; Rodríguez and Joo, 2004; Stinson and Bhat, 2004). In Bogotá, hilly terrains are usually occupied by self-built low-SES neighborhoods. Thus, improving transport accessibility for such areas requires investments beyond improving bicycle infrastructure. Current efforts include the installation of cable cars and increasing the number of bicycle parking facilities near BRT stations to encourage multimodality.

We found that high land use mix (i.e. residential, commercial, etc.) around the location of the trip's destination was negatively associated with bicycle commuting, regardless of gender. Our results differ from previous research in which this factor was found to be positively related to bicycling (Cervero, 1996; Litman and Steele, 2018). This difference might be due to the specific urban configuration of Bogotá in which high land use mix in neighborhoods is common due to the high density of the city (Rodriguez-Valencia et al., 2019). Therefore, the comparisons performed in our analysis are between areas with an already high land use mix against areas with a very-high land use mix (Cervero et al., 2009). Additionally, the proportion of low-stress roads along trip routes had a nonlinear association with bicycle commuting. However, a higher proportion of low-stress roads at trip origin has a positive association with bicycling. Low-stress roads in Bogotá are more frequently present inside residential neighborhoods. Thus, finding low-stress roads along the full commuting route could be challenging, resulting in a need to travel near or on high-stress roads to reach the destination (Huertas et al., 2020). Given that high-stress roads might seem unavoidable in a commuting trip and that such roads discourage less experienced bicyclists (Carvajal et al., 2020), efforts to assure connectivity between bikepaths of lower stress might be useful to attract new riders who may have lower cycling skills (Carvajal et al., 2020).

When analyzing gender-specific factors, we found that age was associated with bicycle commuting. However, the linearly negative relation found in the literature (Moudon et al., 2005; Pucher et al., 1999; Zacharias, 2005) only holds for women. For men, we found a more complex nonlinear relationship between age and bicycle commuting. The propensity for bicycle commuting shows an increase in the range of ages from 14 to 25 among men and then slowly decreases. This result could be explained, in part, because at age 25, income levels among men typically rise (DANE, 2018b), and they usually migrate to other motorized modes of transport, such as a motorcycle or car (Gómez-Gélvez and Obando, 2014). Additionally, the effect of commute distance on bicycle commuting differed by gender. For women, distance is negatively associated, in agreement with preceding findings (Dickinson et al., 2003; Parkin et al., 2008; Stinson and Bhat, 2004; Timperio et al., 2006). Men, on the other hand, are willing to commute for distances shorter than 5 km. This finding suggests that last-mile trips might be successfully promoted among women by increasing access to bike paths close to the most common destinations. For men, bicycle commuting might be encouraged by making bicycle facilities accessible within a 5 km network buffer around administrative centers or bicycle attractor areas in the city where the workforce is concentrated.

When examining the association between bicycle commuting and SES, we found that men who live in very-low to low SES households have a greater tendency to commute by bicycle than those living in a lower-middle SES household. This finding might be explained by the fact that for those with lower income, the available public transportation options are unaffordable. Within this context, the bicycle becomes a more suitable and affordable option. Additionally, middle- and higher-SES men may not be interested in bicycle commuting as they are no longer budget-constrained to using public transport and might gain access to alternative transport modes, such as motorized vehicles, with a better personal cost-benefit ratio compared to bicycling (Gómez-Gélvez and Obando, 2014; Rosas-Satizábal et al., 2020). The motivation for bicycle commuting among those in higher socio-economic groups might come from environmental awareness strategies as discussed by Rodriguez-Valencia, et al. (Rodriguez-Valencia et al., 2019). In addition, having a driver's license for motorcycles or other motor vehicles was negatively associated with bicycle use among men, which is consistent with results shown in the literature (Emond and Handy, 2012; Handy et al., 2014; Orsini and O'brien, 2006; Pucher et al., 1999).

When examining gender and location-specific built-environment factors, we found that having more access to bike paths near the trip destination location is associated with bicycle use for women but not necessarily for men. As expected, improvement in bike path networks is associated with an increment in bicycle commuting (Abraham et al., 2002; Garrard et al., 2007; Hunt and Abraham, 2007; Stinson and Bhat, 2004; Taylor and Mahmassani, 2007). Analyzing female destinations' location should shed light on where to locate the infrastructure to facilitate bicycle commuting among women. In the available literature, having a public transport station near one's home was negatively related to bicycle commuting (Cole-Hunter et al., 2015). Similarly, we identified this association as true for men both in origin and destination for a higher number of BRT stations, but we did not identify the same association for regular bus stops. This difference between BRT and regular buses might be explained by the fact that BRT is faster as it has its own dedicated lane and does not experience traffic delays. Thus, there is additional motivation, especially on longer trips, to choose the BRT over the bicycle, but not necessarily to choose regular buses which must travel within the flow of traffic.

Contrary to other studies, criminal reports within the 500-m buffer surrounding trip origin locations, along trip routes, and at trip destinations were not associated with bicycle commuting. This could be, in part, to the fact that biking for low income population is a necessity instead of a choice or could be related to the level of aggregation of the security data that was available. However, other studies have identified that crime perception was associated with mode choice (Arellana et al., 2020) for the general population and variables such as sexual harassment, safety concerns, and hostility perception towards women in the streets are key for bicycle users (Allen et al., 2018; de la Paz Díaz Vázquez, 2017; Lira, 2020; Montoya-Robledo et al., 2020; Orozco-Fontalvo et al., 2019). Thus, participatory methods involving women's security perceptions in urban city planning is key and security perception data in cities might facilitate the assessment of this factor in future analysis.

Cities with a recently expanded and extensive bicycle infrastructure or aiming to promote active transportation modes should consider women's destinations and the surrounding infrastructure to increase women's share. This study highlights the need to include gender-specific perspectives in the Global South cities to avoid mobility planning that responds solely to men's needs (Montoya-Robledo et al., 2020) and reinforces the unequal gender labor distribution (Singh, 2020). More specifically, during the COVID-19 response, several strategies have been implemented globally towards biking accommodation (Combs and Pardo, 2021; Singh, 2020). Thus, urban planners should, more than ever, involve gender-specific and location-specific factors influencing bicycle commuting to enhance their public policy frameworks. In this way, it facilitates access to bicycle use in women and potentially bridges the gender gap in bicycle commuting. Interesting initiatives such as the Gender and Mobility Strategic Plan, an initiative of the local Mexican government, seem promising (Lira, 2020).

This study's limitations include the undercounting of bicycle trips given that Bogotá's travel survey is focused on a single day rather than in a travel diary that would be more likely to capture all trips, including bicycle trips. However, Bogotá's travel survey is the most reliable source that accounts for all trips with representativeness at the city level. We could not assess the built-environment factors of bicycle commuting longitudinally because there are no origin and destination coordinates available in previous surveys. 2015 Bogotá's travel survey was the first mobility source with this level of disaggregation.

## 6 Conclusion

This study analyzed the trends in the number of bicycle commuters in Bogotá for 12 years and found a persistent and widening gender gap in bicycle commuting. Higher rates of growth in men commuting by bicycle reveal an important gender disparity and opportunity to identify strategies for increasing female bicycle commuting. Additionally, we found that associations between socio-demographic and built-environment factors and bicycle commuting differ by gender and trip stage.

Results suggest that approaches to promote bicycle commuting in Bogotá should be developed with intersectoral, participatory and gender perspective aiming to understand the mobility of care in the city. The investment in bicycle infrastructure at common destinations within the city and the promotion of bicycle commuting to female students might be examples of targeted strategies for women. Additionally, discouraging car use and lowering the level of stress on bicyclists on roads near the most common trip starting points (using strategies such as lowering speed limits and traffic calming) may help increase overall bicycle use. The same can be said of implementing strategies to create better connectivity between low-stress roads on commonly used bicycle commuting routes. Mobility surveys in LAC, should include origin and destination data to be able to conduct location-specific and sex stratified analysis. Our methods could serve as a tool to guide and plan potential policy interventions aiming to promote bicycle commuting with differential and gender-inclusive approaches.
